# Pervasive Modulation of Obesity Risk by the Environment and Genomic Background

**DOI:** 10.3390/genes9080411

**Published:** 2018-08-14

**Authors:** Sini Nagpal, Greg Gibson, Urko M. Marigorta

**Affiliations:** Center for Integrative Genomics, Georgia Institute of Technology, Atlanta, GA 30332, USA; sini.nagpal@gatech.edu (S.N.); ggibson.gt@gmail.com (G.G.)

**Keywords:** diseases of affluence, body mass index, gene-by-environment interactions, genome-wide association studies (GWAS), polygenic scores (PGS), epistasis, allele expressivity, UK Biobank

## Abstract

The prevalence of the so-called diseases of affluence, such as type 2 diabetes or hypertension, has increased dramatically in the last two generations. Although genome-wide association studies (GWAS) have discovered hundreds of genes involved in disease etiology, the sudden increase in disease incidence suggests a major role for environmental risk factors. Obesity constitutes a case example of a modern trait shaped by contemporary environment, although with considerable debates about the extent to which gene-by-environment (G×E) interactions accentuate obesity risk in individuals following obesogenic lifestyles. Although interaction effects have been robustly confirmed at the *FTO* locus, accumulating evidence at the genome-wide level implicates a role for polygenic risk-by-environment interactions. Through a variety of analyses using the UK Biobank, we confirm that the genomic background plays a major role in shaping the expressivity of alleles that increase body mass index (BMI).

## 1. Introduction

Non-communicable diseases such as diabetes, cancer, or cardiovascular disease, have become the leading cause of death across most human populations [1]. The prevalence of these maladies increased steadily over the course of the 20th century in high-income societies, prompting the label Western disease, or disease of affluence [2]. These umbrella terms are used to imply that the global epidemiological shift arises from changes in habits and lifestyle associated with improved living conditions following economic progress. As of 2016, half of the top 10 causes of death in lower to middle income countries are non-communicable diseases such as stroke and diabetes [3]. Although the suddenness of the change implies that surges in prevalence are influenced by new environmental risk factors, heritability analyses indicate that genetic variation continues to play an important role in susceptibility to modern disease [4].

Obesity is a central trait in the epidemics of affluence. The current sedentary nature of human behavior leads to higher body mass index (BMI) since caloric intake is not offset by exercise. For instance, the average US citizen walks ~50% less steps than Amish individuals who follow a traditional farming lifestyle, and this corresponds to an excess retention of 300 kcal/day [5]. These changes are leading to massive increases in obesity rates, having for example risen from 10 to 25% in England in just the last two decades [6]. Remarkably, besides moving the average BMI ‘towards the right’ of the distribution, under the current obesogenic lifestyle the whole distribution has also flattened towards the right. For instance, after strong westernization the average BMI in Korean men increased by 0.06 BMI units per year in the 1998–2014 period. However, the increases have been much greater at the top, with up to 40% larger increasing rates at the 95th percentile of the distribution [7]. Similar steady increases in the mean and variance of BMI distributions are occurring worldwide [8].

Although there is little doubt that alterations in lifestyle account for the increasing prevalence of obesity, there is however conflicting evidence about the participation of gene-by-environment (G×E) interactions and whether heritability of this trait has been affected. A strongly held viewpoint is that the effect of the environment is simply additive. Interaction effects can be considered at three levels: the individual genotype (at the level of single single nucleotide polymorphisms, SNPs), polygenic effects (the summation of multiple risk factors), and the whole genome common variants (SNP-heritability). Evidence for individual genotype-by-environment interactions in human cohort studies is very sparse, despite the well-known example of the largest risk factor for obesity, the *FTO* locus, robustly shown to have a stronger influence as BMI increases [9,10]. No such interactions have been described for height, or for most analyses of disease susceptibility [11,12]. However, recent work has begun to show that polygenic scores (PGS, also known as polygenic risk scores or PRS in the context of disease) may vary in the magnitude of variance explained across strata of individuals who for example have different environmental exposures such as smoking, physical activity, or sugary beverage consumption [13,14,15,16], or across birth cohorts [17]. Recently, Abadi et al. [18] extended this concept by demonstrating that PGS for BMI increase the proportion of variance explained in higher deciles of BMI. Such results might be expected to lead to differences in heritability over time as trait frequency distributions change, but a comprehensive review of the evidence in the UK Biobank found only limited evidence for this in relation to BMI, and none for height [19]. Similarly, meta-analyses of large twin cohorts have found that heritability of BMI has mostly remained constant since at least the second half of the 20th century [8,20]. Given the large increase in environmental variance, constant heritability—or even marginally larger in some cases—implies increased genetic variance contributions as well. Interpretation of conflicting results is complicated by issues related to appropriate methods for scaling the variances [21].

The possibility that GxE effects are more pervasive than currently accepted raises concerns over the limitations of current genome-wide studies to uncover them, and subsequently to completely characterize the genetic architecture of traits [22,23]. Genome-wide association studies (GWAS) usually involve meta-analyses of large cohorts, often from different countries, because vast sample sizes are needed to detect susceptibility alleles with robust statistical support. Unfortunately, meta-analyses of summary statistics are not able to account for the effect of environmental lifestyle covariates. Furthermore, they detect only average effects and implicitly assume that allelic effects are similar in all individuals with the same genotype, necessarily missing the true heterogeneity in effects that results from G×E interactions. We have also argued with the support of simulation studies that if pervasive, interaction effects hinder the statistical power to even detect marginal effects in the first place [22]. More focused efforts to detect polygenic score-by-environment interactions hold the key for understanding the genetic architecture and true impact of modern lifestyle on the diseases of affluence.

We used the UK Biobank to test two hypotheses related to the question of whether interaction effects make a meaningful contribution to the genetic architecture of a complex trait such as BMI. We take advantage of the very large size of the study and the extensive mapping of BMI-associated variants from independent studies to ask how genetic effects vary as a function of obesity and genetic background. Specifically, we evaluated the evidence (i) that BMI-enhancing interactions (G×E) extend across SNPs that do not achieve genome-wide significance in GWAS, and (ii) that the polygenic background alters the effect of known BMI-increasing alleles in an epistatic gene-by-gene (G×G) fashion. We infer that both phenomena, namely G×E and G×G, do contribute substantially to the genetic basis of weight control. Using BMI as a trait model, these results are consistent with the inference that modern environment plays a key role in shaping the genetic basis of diseases of affluence.

## 2. Materials and Methods

### 2.1. Processing the Genetic and Phenotype Data From the UK Biobank

The UK Biobank is a large population-based cohort study that between 2006 and 2010 recruited over 500,000 adults living <25 miles from 22 assessment centers across the UK [24]. The participants, aged 40–69 at recruitment, completed baseline questionnaires about lifestyle, medical history and general health, as well as providing biological samples that were stored for future analysis. In 2015, an interim release of genomic assays for 150,000 individuals was made available for research. Samples with sex mismatches, excessive missing genotypes, outlier heterozygosity rates, or high relatedness (third degree or closer) were filtered out centrally by the study [25]. The participants′ DNA was genotyped on two arrays, UK Biobank BiLEVE Axiom® array and UK Biobank Axiom® array (Affymetrix, Santa Clara, CA, USA) that have >95% shared variants, for a total of 805,426 SNPs that passed batch quality control in up to approximately 150,000 individuals. From this release, we selected 119,841 unrelated participants of self-identified White British descent whose European ancestry had been centrally confirmed through principal component analysis (PCA). For each participant, we retrieved age, gender, 10 first principal components to control for population structure, as well as height and weight from first examination to calculate BMI (by dividing the weight (in kg) by the square of the height (m)). In May 2017, a new release with corrected imputation data for ~96 million markers was made available for the full UK Biobank dataset [25]. To increase marker availability in our study, we downloaded this version of the imputed data (named v3) for the previously ascertained 119,841 individuals. After selecting only bi-allelic variants with imputation score >0.9, minor allele frequency (MAF) >1%, Hardy-Weinberg equilibrium (HWE) *p* > 10^−10^ and <5% missing rate, a total of 8,063,507 SNPs remained available for analysis. All selected participants presented <1% missing genotyping rate.

### 2.2. Stratified GWAS

Besides classical GWAS using all the individuals, for initial exploratory analysis we performed BMI-stratified GWAS to inspect whether the effect of common variants with at least 1% MAF varies according to the BMI category of individuals. Specifically, the selected participants with genetic data available were assigned into four categories based on their BMI values (original range 12 to 65): underweight (BMI < 20, *n* = 2685), normoweight (BMI 20–25, *n* = 35,955), overweight (BMI 25–30, *n* = 51,132), and obese (BMI > 30, *n* = 30,069). For this analysis we used the ‘—linear—covar’ routine available in the whole-genome analyses toolset PLINK [26] to perform linear regression separately on each of the four subgroups of individuals. Along with age and gender, to correct for population substructure we also included the first ten principal components of genetic variation as covariates.

### 2.3. Calculation of Genetic Risk Scores

Given the small effect size of variants discovered by GWAS, it is common practice to pool together the effects of all known variants into PGS that summarize the genetic liability for each individual. To calculate PGSs based on variants associated with BMI, we used the association results of the GWAS for BMI by the GIANT consortium [27]. This meta-analysis used genetic data for ~339,000 individuals from 125 studies to discover 97 SNPs associated with BMI at genome-wide significance and up to 289 variants with suggestive association (*p* < 10^−5^).

In total, we calculated six different unweighted PGSs using the ‘—score’ routine available in PLINK. First, to replicate the findings by Abadi et al. [18], we calculated a classical PGS using the 289 variants reported by GIANT as having strong evidence of association (*p* < 10^−5^; gathered from Supplementary Table S4 in [27]). This analysis included 283 SNPs because two variants were missing and four were pruned out due to high pairwise linkage disequilibrium (LD; Supplementary Table S1).

Next, to explore whether environment-dependent variation in effect size extends to SNPs that are not genome-wide significant, we calculated four further PGSs including variants associated with BMI at different suggestive significance thresholds in the GIANT study: (i) 230 SNPs at 10^−7^ < *p* < 10^−5^, (ii) 2418 SNPs at 10^−5^ < *p* < 10^−3^, (iii) 9975 SNPs at 10^−3^ < *p* < 0.01, and (iv) 32,423 SNPs at 0.01 < *p* < 0.05. As a negative control, we included a sixth PGS based on 39,108 common SNPs (MAF > 5%) that are not associated with BMI according to the GIANT study (*p* > 0.95).

To ensure that these PGSs were not simply capturing the effects of stronger nearby signals from the GIANT study, before selection we previously removed all SNPs correlated (LD *r*^2^ > 0.2) with any of the 283 SNPs used in the aforementioned PGS. Moreover, to avoid overfitting due to variants with similar *p*-value that lie in the same LD block, these PGSs only include SNPs that are not in strong linkage disequilibrium among each other. Specifically, we used ‘—indep-pairwise 10 5 0.5′ command in PLINK to filter out variants at LD *r*^2^ > 0.5 in 10kb windows with a 5-SNP sliding step. Finally, to avoid recapturing the signal that other scores independently account for, progressively for each PGS we obtained the residuals after regressing out those PGSs based on SNPs with stronger *p*-values of association with BMI. For instance, for the case of the PGS based on SNPs with 10^−5^ < *p* < 10^−3^ in GIANT, we moved forward for analysis the residuals after fitting the two previous PGSs based on SNPs with stronger association with BMI, namely 10^−7^ < *p* < 10^−5^ and the abovementioned 283-SNP one. This fitting step ensured that all the residuals used in the analyses were uncorrelated among each other, and therefore the effects estimated in the quantile regression (see Section 2.4) truly correspond to the independent signal arising from the SNPs included in the PGS under consideration.

As in Abadi et al., we used the unweighted version of each PGS—namely the number of BMI-increasing alleles per individual—as a baseline for our analyses. However, we also inspected the patterns observed with weighted PGS calculated using the betas from the GIANT study as weights. A list of SNPs included in each of the PGSs, along with the information to calculate PGS with PLINK, is available in Supplementary Table S2.

### 2.4. Quantile Regression Analyses

Following the procedures used by Abadi et al., we performed quantile regression analyses to explore whether the effect of each of the six calculated PGSs varies across the distribution of BMI in the UK Biobank dataset. The rq function available in the R package *QuantReg* [28] was used to fit a linear model of BMI ~ PGS through quantile regression at each BMI decile, and generated linear plots for the series of ordinary least squares (OLS) estimates for each PGS (average estimate and confidence intervals for each fit were extracted with the summary function). Age, gender, and the first ten principal components of genetic variation were included as covariates for each regression. Note that this procedure does not scale the variance in each decile, so there is some expectation that genetic effects, reported here as beta values from the slope of the regression when using unweighted PGS, increase as the trait increases. These β can be interpreted as the substitution effect of the increasing allele of a genotype for the alternate allele. We repeated the same analyses using the weighted PGS, which were scaled prior to quantile regression and therefore the β corresponds to the estimate of the effect on BMI per standard deviation of PGS. Finally, to correct for the global mean-variance relationship, we also reran the analyses for each PGS after rank inverse normal transformation of BMI.

### 2.5. Expressivity Analyses

We performed allele expressivity analyses to determine whether the effect of each variant associated with BMI changes according to the genetic background. For each variant, we used the lm function available in the R environment to fit ten different linear models (i.e., BMI ~ SNP), each restricted to 11,927 individuals in each decile of the PGS based on GIANT SNPs introduced above (we used 276 SNPs after removal of 7 variants with MAF < 5%). The estimates from each decile were used to calculate a global slope for each SNP, obtained through regression of PGS deciles on the 10 slopes. Next, we counted the proportion of slopes (one for each of the 276 SNPs) that were positive (i.e., the effect of the SNP on BMI scales positively with increasing PGS) and used the binomial test function available in R to test its statistical significance.

## 3. Results

There is increasing evidence that genetic variants involved in susceptibility to the diseases of affluence are affected by a myriad of perturbed environmental factors associated with modern lifestyle. Using BMI as a model trait, we analyzed genetic data for approximately 120,000 individuals from the UK Biobank to carry out three complementary analyses aimed at garnering evidence that the genetic and environmental backgrounds alter the effects of BMI-associated variants.

We first performed a preliminary GWAS for additive effects using all the selected individuals. In total 49 independent regions achieved genome-wide significance, with the strongest signal observed at the *FTO* locus (rs11642015, *p* < 10^−79^). We next compared the effect size at 283 variants discovered by the GIANT consortium [27] with the estimates from our cohort. Remarkably, 209 (73.9%) and 59 (20.8%) of the variants replicated at *p* < 0.05 and *p* < 10^−5^, respectively, in the UK Biobank cohort (~14 and 0.003 expected randomly, Supplementary Table S1), with an overall strong correlation in effects sizes at these 283 variants (Pearson′s *r* = 0.89, *p* < 10^−16^, Supplementary Figure S1). This classical GWAS result using all individuals confirms that the UK Biobank is a reasonable cohort to study BMI genetics.

### 3.1. Stratified GWAS Suggests Stronger Genetic Effects in Obese Individuals

**Figure 1:**
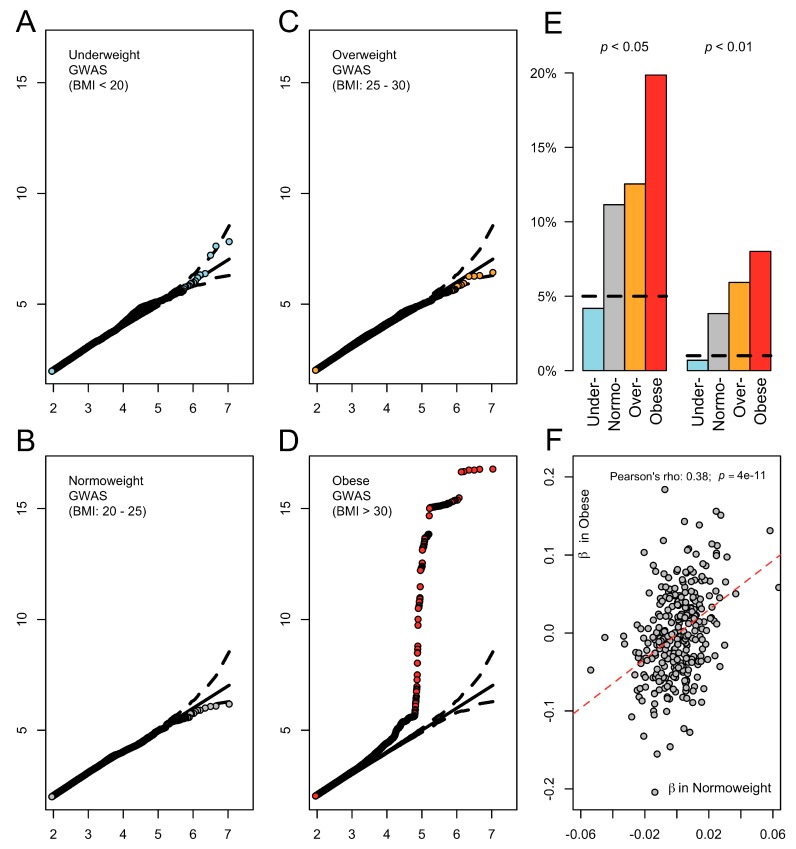
The genetic control of body mass index (BMI) differs across body fat categories. (**A**–**D**) Q-Q plots depicting genome-wide association results for stratified-genome-wide association studies (GWAS) for each BMI category. *Y*-axis displays the -log_10_ of the *p*-value of association derived from linear regression (see Methods Section 2.2 *Stratified GWAS*). (**E**) Barplots showing the percentage of the 283 BMI-associated single nucleotide polymorphisms (SNPs) (see Methods Section 2.3 *Calculation of Genetic Risk Scores*) that pass nominal significance (*p* < 0.05 and *p* < 0.01 are depicted). With the exception of the underweight sub-group, all the other three BMI subgroups harbor an excess of positive replications of the GIANT signals, implying an excess of real effects. The horizontal dashed line corresponds to random expectation. (**F**) Scatterplot of the β in normoweight and obese subgroups for the same SNPs.

After ensuring the reliability of the data, we started by performing four BMI-stratified GWAS, one for each of the main categories that are typically used to classify individuals according to their BMI (see Methods Section 2.2 *Stratified GWAS*). For each BMI subgroup, Figure 1A–D show Q-Q plots depicting the genome-wide distribution of *p*-values for additive effects at each of the ~8 million SNPs available in the UK Biobank. At first glance, there is a stark absence of genome-wide significant effects for GWAS since only for the GWAS restricted to obese individuals is there a single strong association peak detected, which corresponds to the well-known obesity-driven signal at the *FTO* locus (top SNP rs11642015, *p* = 2 × 10^−17^). This signal, for instance, weakens progressively in leaner individuals, with *p* = 8 × 10^−4^, *p* = 0.15 and *p* = 0.50 at the peak SNP in over-, normo-, and underweight categories, respectively. An exploration of the effects at the 283 BMI-associated SNPs from GIANT renders a similar pattern whereby the GWAS on the obese subgroup shows the strongest enrichment for replicated signals (Figure 1E). Similarly, the betas at these variants are correlated between normoweight and obese subgroups (Pearson’s *r* = 0.38, *p* < 4 × 10^−11^), but again with stronger estimates for the latter group (Figure 1F). Besides confirming once again the daunting number of samples that are needed to study the genetics of BMI, these results suggest that the genetic control of weight differs between lean and obese individuals.

### 3.2. BMI-Related Expressivity of Polygenic Effects Extends to Variants Below the GWAS Threshold

Next, we set to determine the evidence for variation in the effect of weight-controlling variants across the distribution of BMI. Recently, Abadi et al. [18] demonstrated that 9 of 37 BMI-associated SNPs have effects that increase steadily in higher BMI percentiles in a compendium of North American studies. Additionally, the β associated with a PGS derived from the 37 SNPs increased with BMI, a pattern which is consistent with extensive interactions with the obesogenic environment. To confirm and extend this observation in the UK Biobank, we calculated five different PGSs based on increasingly larger numbers of genetic variants with progressively weaker statistical association with BMI, as well as a sixth one based on non-associated SNPs that serves as a negative control (see Methods Section 2.4 *Quantile Regression Analyses*). This allowed us to explore whether the effects of PGS×E interactions extend to the whole genome in an infinitesimal fashion.

**Figure 2:**
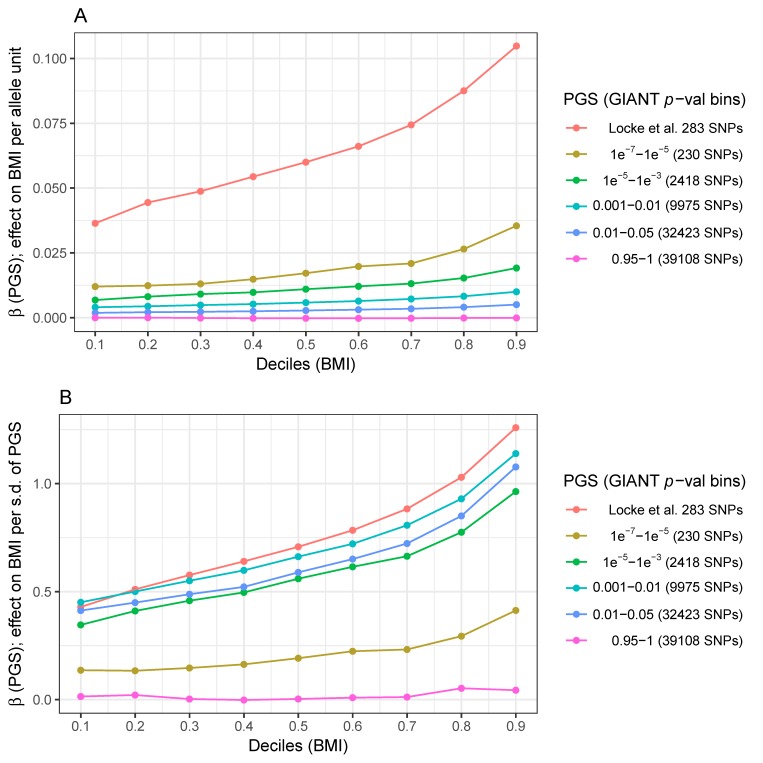
The effect of polygenic scores for BMI varies across the BMI distribution. Summary of the effects that six unweighted polygenic scores (PGS) have by decile of the BMI distribution in the UK Biobank cohort. The ordinary least squares (OLS) estimates of the effect on raw BMI per allele and per standard deviation of the weighted PGS (*Y*-axis for panel **A** and **B**, respectively) are plotted against the corresponding decile of BMI (*X*-axis). As noted in the legend, each PGS was calculated by combining SNPs associated with BMI at different statistical thresholds in the GIANT consortium meta-analysis (see Methods Section 2.4 *Quantile Regression Analyses*). A list of SNPs used for calculating each PRS, including risk allele, are available in Supplementary Table S2.

A series of OLS models were fit for each PGS based on different GWAS *p*-value bins, namely 10^−7^ < *p* < 10^−5^ (230 SNPs), 10^−5^ < *p* < 10^−3^ (2418 SNPs), 10^−3^ < *p* <0.01 (9975 SNPs), 0.01< *p* < 0.05 (32,423 SNPs), and lastly, *p* > 0.95 (39,108 SNPs). Figure 2A plots the β estimates of the substitution effect of each unweighted PGS on BMI in deciles of the BMI distribution. Confirming the results of Abadi et al. using cohorts unrelated to the UK Biobank, the effects of the PGS based on 283 GIANT SNPs increase significantly across the BMI distribution with effects per allele that are over two-fold stronger in the highest vs. lowest BMI decile.

We extend their result with the observation of a similar pattern for less strongly associated SNPs that are nominally significant but do not pass genome-wide significance thresholds and hence are not formally associated with BMI. The PGS based on 230 further independent variants at 10^−5^ < *p* < 10^−7^ shows a clear pattern whereby the effect of the PGS is also enhanced for overweight and obese individuals. This pattern extends albeit progressively less strongly to the polygenic scores using SNPs that are only nominally associated with BMI (from 10^−5^ < *p* < 10^−3^ up to 0.01 < *p* < 0.05; plots specific for each PGS including also the 95% confidence interval of the estimates are available in Supplementary Figure S2). As expected, the PGS based on SNPs not associated with BMI presents a flat distribution of OLS estimates. The corresponding analyses using weighted PGSs show similar patterns whereby the penetrance of variants increases as a function of BMI (Figure 2B and Supplementary Figure S3). Interestingly, the effects of these weighted PGSs on BMI (~0.5 s.d. units) are almost as large as that of the 283 BMI-associated SNPs discovered by GIANT. These analyses confirm that, thanks to their abundance, SNPs weakly associated with BMI play a major role in the architecture of this trait.

The mean-variance relationship in BMI can induce larger within-decile phenotypic variation for the highest BMI deciles. This phenomenon could lead to larger β estimates that, rather than implying stronger genetic effects, may simply reflect differences in the scale. To explore this possibility, we fitted the same series of OLS models using inverse normalized instead of raw BMI values. Although global control of the mean-variance relationship necessarily removes part of the signal for the PGSs based on SNPs weakly associated with BMI (Supplementary Figure S4), an exploration of each PGS individually confirms that genetic effects are stronger in obese individuals (Supplementary Figures S5 and S6).

These results provide evidence that a large number of variants, well beyond the limited set of genome-wide significant SNPs discovered by the GIANT consortium, exert a stronger effect on BMI in individuals who are exposed to a stronger obesogenic environment.

### 3.3. Gene-by-Polygenic Scores (G×PGS) Analysis Hints at Epistatic Interactions Shaping Genetic Effects for BMI

**Figure 3:**
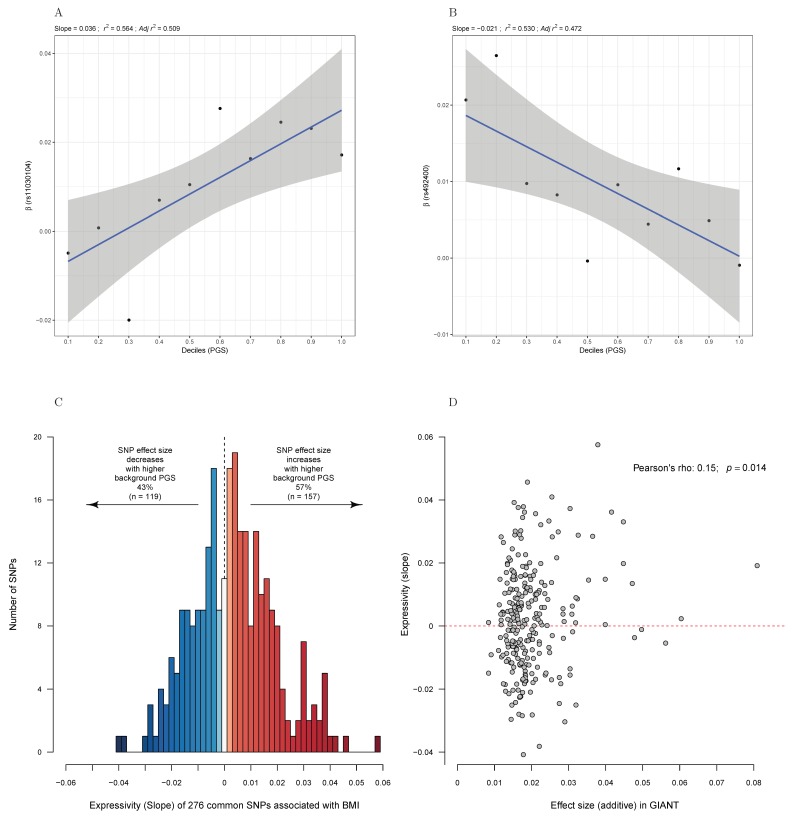
Allele expressivity of BMI variants changes according to the genetic background. The effect of BMI-associated variants tends to increase in more obesogenic genetic backgrounds. (**A**) Effect of rs11030104 on BMI for different deciles of a PGS combining the allele counts per individuals for 276 common variants associated with BMI (see Methods Section 2.5 *Expressivity Analyses*). (**B**) The corresponding allele expressivity slope for rs492400 is negative, indicating that the BMI-increasing allele does indeed lower it in individuals with a strong obesity-predisposing genetic background. (**C**) Histogram depicting the estimate of allele expressivity slopes for the 283 SNPs associated with BMI. The significant trend towards positive slopes (in red) implies that variants associated with BMI tend to exert stronger effects in individuals with higher PGS. (**D**) Scatterplot depicting for each variant the effect size on BMI in the GIANT study (*X*-axis) against the allele expressivity slope observed in the UK Biobank (*Y*-axis). These variables are positively correlated (Pearson correlation coefficient *r* = 0.15, *p* = 0.014), particularly at variants with stronger effects on BMI.

Finally, we performed a global test to determine whether the 283 BMI-associated SNPs discovered by the GIANT consortium are affected by a form of higher-order epistatic interaction with the genetic background. To increase reliability of the analyses, we only included those variants with MAF > 5% (*n* = 276). Specifically, for each variant we calculated its additive effect on BMI using individuals from each decile of the 276-SNP PGS (see Methods Section 2.5 *Expressivity Analyses*) and plotted the slope of the regression. In Figure 3A,B, the examples from two SNPs are shown. For instance, the effects of rs11030104 (*BDNF*) on BMI increase in individuals with higher genetic predisposition to BMI. Conversely, the opposite pattern is observed for rs492400 (*USP37*). Since there is low power given noisiness of estimates for SNPs that individually explain of the order of 0.1% of the variance for BMI, we report the overall distribution of slopes. For 57% (*n* = 157) of the 276 BMI SNPs with MAF > 5%, the trend is for expressivity to increase as the PGS decile increases (*p* = 0.026, Binomial test, Figure 3C). This figure increases to 68% when restricting to SNPs that are genome-wide significant in the full UK Biobank cohort (21 out of 31, *p* = 0.071). Furthermore, Figure 3D shows that the trend towards increased effect size along with genomic susceptibility to high BMI is particularly strong for SNPs with larger effects on BMI. These results suggest that not only do genetic influences on weight gain increase in obesogenic environments, but also that they are increased in obesogenic genetic backgrounds.

## 4. Discussion

The analyses reported here add to a growing body of evidence that genetic influences on BMI/weight gain are collectively influenced by genetic and environmental interactions. Despite the scant evidence for individual G×E interactions at genome-wide significance levels, polygenic score interactions are detectable. A major reason for the low evidence for G×E is the small effect sizes: if main effects are only detected in samples of 50,000 individuals or more, and interactions explain a fraction of the variance, then it is not surprising that individual SNP G×E are for the most part undetectable. There is also compelling theory arguing that individual G×G between pairs of common SNPs should be rare [29], but this does not preclude interactions with the genetic background.

For cumulative interaction effects to be observed, it is necessary that the effects are consistently in the same direction. This seems to us reasonable: if an obesogenic environment such as sedentary behavior or a high-fat diet causes the effect at one SNP that tends to increase BMI to increase further, then it should act similarly on most other weight-gain associated alleles. The authors of the omnigenic model noted that a few percent change in allele frequency at even a minority of the over 100,000 variants now expected to be associated with any complex trait could translate into highly significant divergence among populations [30]. Similarly, barely detectable changes in effect size in obesogenic environments could cumulatively result in the types of change in trait distribution that we see for BMI, and by extension risk of many common diseases.

Whether or not this is the case remains open to interpretation. One objection to the PGS×E approach is that it does not account for the expected scaling of variance with the mean. On this strict interpretation, G×E should only be considered to be statistical interactions if they remove, induce, or reverse the sign of an allelic effect, namely if they cause so-called crossing of line means between environments. A pervasive tendency for effects to simply increase would not be considered an interaction effect, since scaling of the means can always remove the effect statistically. Yet, the exercise of scaling the variance in each decile, for instance, would necessarily entail a reduction in the effects of any active interaction and hence the power to detect them. Remarkably, we show that evidence for differential penetrance of BMI-associated variants still remains after accounting of the mean-variance relationship at the global level through inverse normal transformation of BMI. More work, possibly based on simulations, is needed to clarify this point. In any case, we would argue that, biologically, increased genetic effects are contributing to population-wide increased BMI whether or not they are significant after scaling. That is to say, G×E involving a simple increase in effect size are meaningful interactions.

An ample presence of interaction effects shaping the architecture of Western disease fits with the expectations under the decanalization model [4]. Any process that leads to a global increase in genetic effect sizes in a new environment is changing the genetic architecture consistent with release of genetic variation. Under long-term stabilizing selection, the genetic variance in a population is expected to decrease, ensuring that individuals tend to be closer to the mean than they would be under purely additive conditions. Since the contemporary human lifestyle is new, the conditions under which alleles act may have changed such that genetic effect sizes change globally, specifically increasing the genetic variance. There is as yet little empirical evidence that this process is increasing the prevalence of disease, with the exception of type 2 diabetes risk, which is itself a function of BMI. Blood glucose levels vary as a function of two traits: insulin release (by the pancreas) and insulin sensitivity (by the target tissues). As described by Kahn et al. [31], canalization of glucose regulation renders a hyperbolic function in which perturbations in one trait can be easily offset by concurrent changes in the other trait (e.g., higher insulin release can compensate for impaired glucose tolerance). A systematic review of the literature has corroborated this prediction: normo-glycemic cohorts are positioned along the predicted canalized stabilization points, whereas individuals with impaired management locate outside the curve [32]. Interestingly, there are ethnic differences in the stabilization points of Africans, East Asians and Europeans, the latter being particularly placed in the most robust optimum [32]. This finding could explain the worldwide patterns of type 2 diabetes prevalence, which are particularly high in non-European populations [33].

Several limitations can be identified in this work. For instance, most of the evidence we present, such as the G×PGS analyses, is based on global tendencies. Only larger datasets with improved genomic coverage will gather clear-cut evidence at the level of individual SNPs. Related to this, our indirect approach permits the inference that interactions are occurring, but only targeted inclusion of environmental data will demonstrate whether the source of these interactions are related to the current obesogenic lifestyle or other environmental factors. Moreover, using large cohorts entails risks related to hidden biases. The UK Biobank harbors sample bias, for instance, because the participants are known to have lower BMI than the general population. This would affect the spectrum of environmental variance available for G×E studies, and possibly limit statistical power. More worryingly, hidden unaccounted genetic structure could potentially affect PGS analyses, as it was recently shown for the relationship between PGSs by recruitment center and several metabolic phenotypes [34]. Specifically, the presence of hidden population structure could correlate with regional differences in BMI and therefore inflation of the differences in penetrance that we report. Additionally, obese individuals may suffer from an elevated proportion of rare alleles whose effects are cumulatively likely to be tagged by common variants, and it is difficult to judge the magnitude of any inflation due to this effect. More work is needed to clarify its potential impact on GWAS, even more so for studies looking for gene-gene and gene-environment interactions that can only be discovered through polygenic scores calculated in large cohorts such as the UK Biobank.

Yet, overall our results lend support for interactions between genes and environments playing a major role in the architecture of complex traits. This work is embedded in the strong wave of studies that are uncovering the important role that G×E (and potentially G×G) play in BMI. Considering that being obese increases the life-time risk to develop disease, gaining insights into the role of interactions will lead to improved understanding of the genetic bases of modern disease.

## Supplementary Materials

The following are available online at http://www.mdpi.com/2073-4425/9/8/411/s1, Figure S1. Correlation in effect sizes for 283 BMI-associated variants discovered in the GIANT meta-analysis and the UK Biobank cohort. Figure S2. OLS estimates of the effects on raw BMI for each unweighted PGS. Figure S3. OLS estimates of the effects on raw BMI per standard deviation of each weighted PGS. Figure S4. OLS estimates of the effects of unweighted and weighted PGS on inverse normalized BMI. Figure S5. OLS estimates of the effects on inverse normalized BMI for each unweighted PGS. Supplementary S6. OLS estimates of the effects inverse normalized BMI for each weighted PGS. Table S1: Association data for the 283 BMI-associated SNPs discovered by the GIANT consortium. Table S2: Input files used for calculating PGS.
