# Supplementary figures and images for: Pervasive Modulation of Obesity Risk by the Environment and Genomic Background
Source: Genes (Basel). 2018 Aug 14;9(8):411. doi: 10.3390/genes9080411 (PMC6115725; doi:10.3390/genes9080411)

Beta in UK Biobank

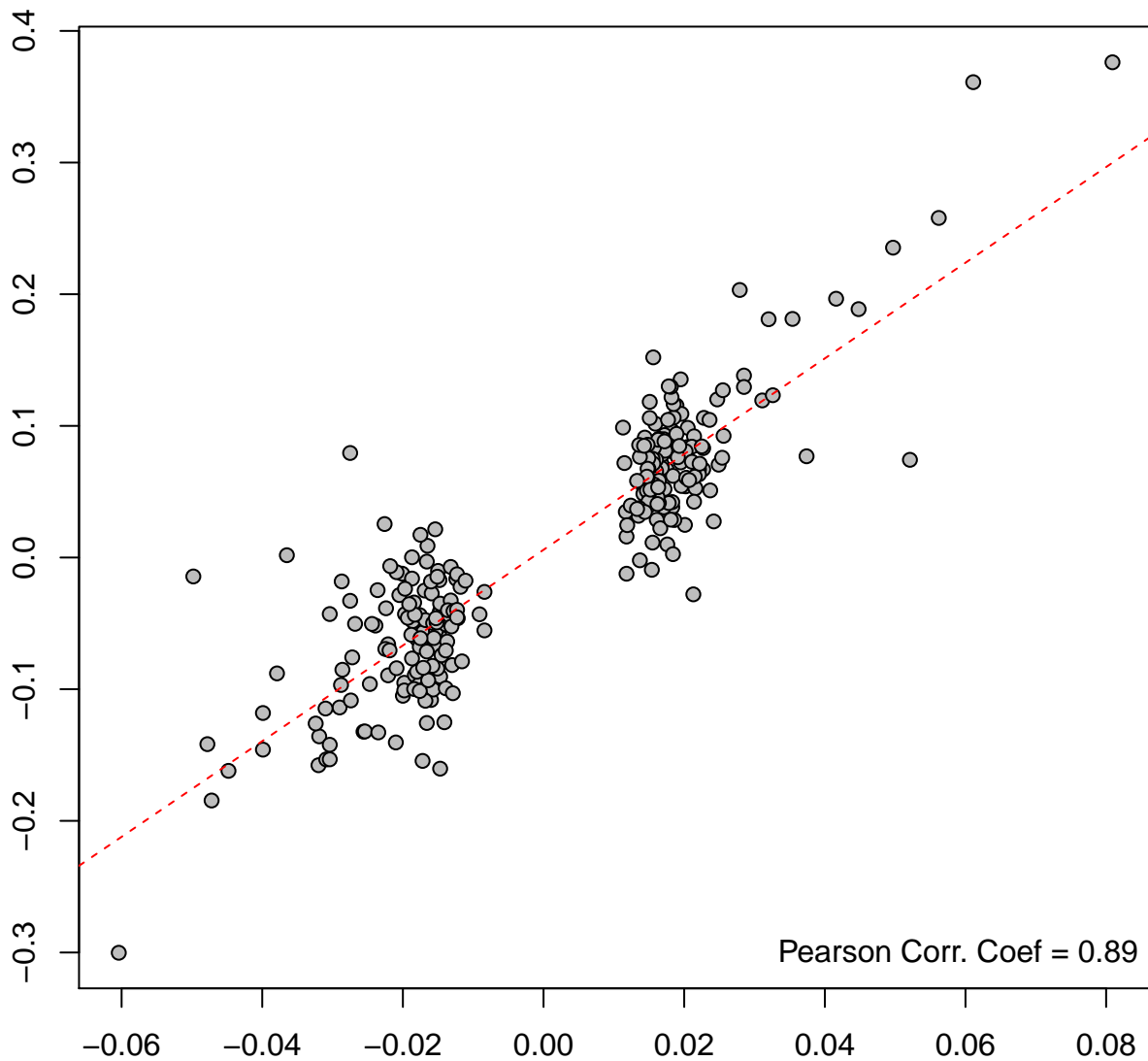

Beta in GIANT (Locke et al., Nature, 2015)

Supplement: Supplementary file 1 [file genes-09-00411-s001.zip › SupplementaryFigure1.pdf]

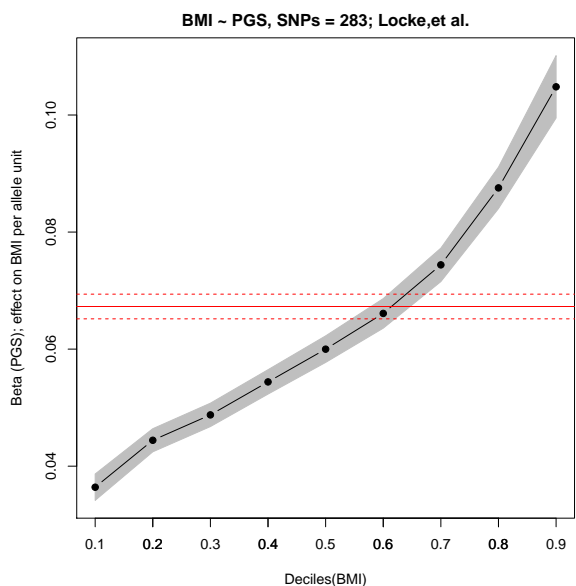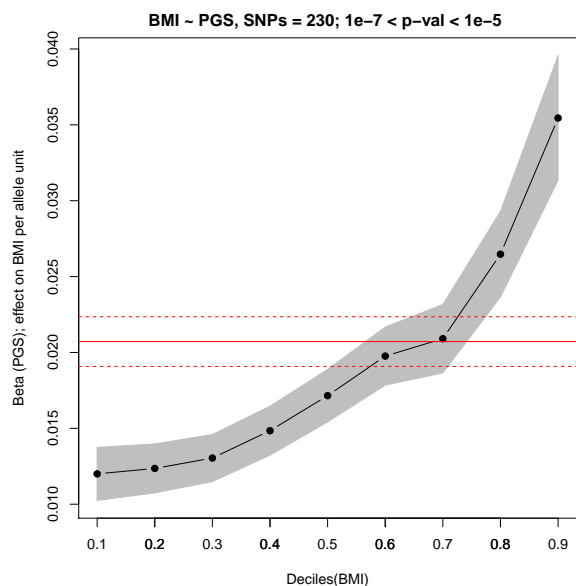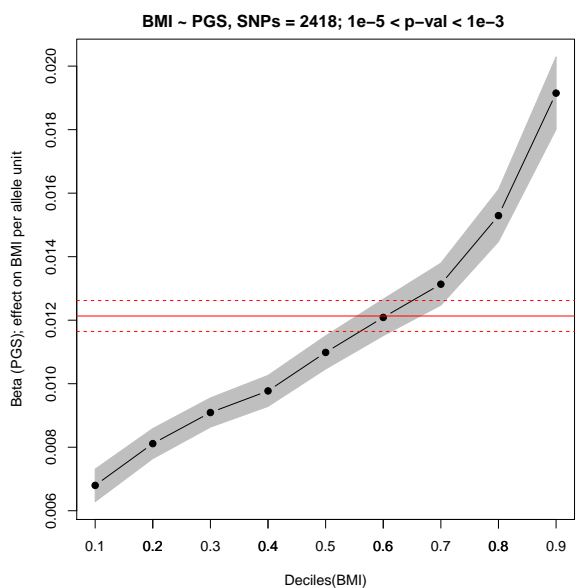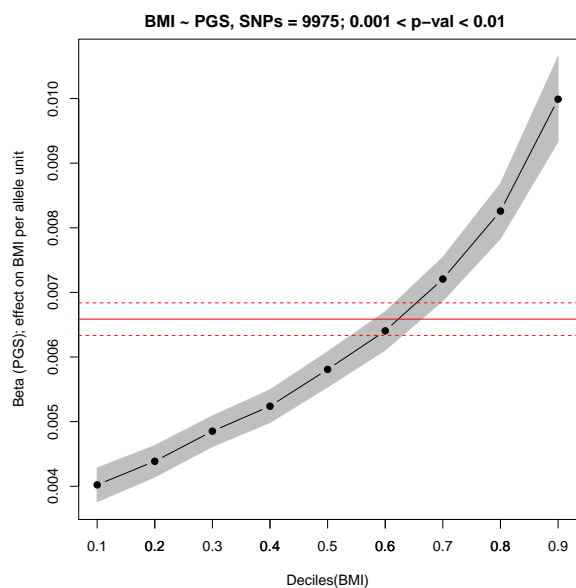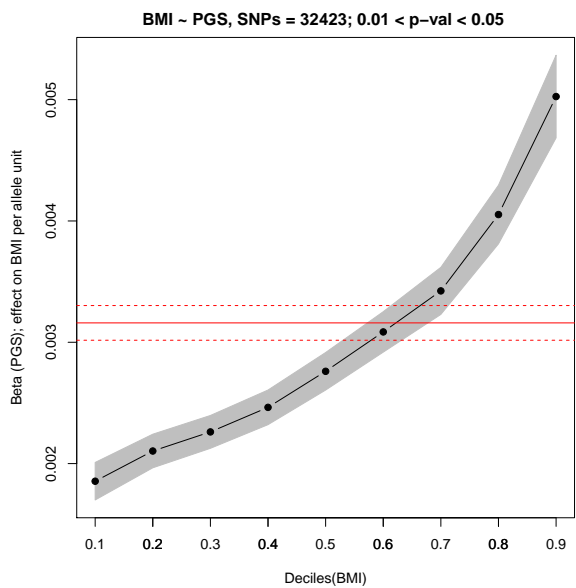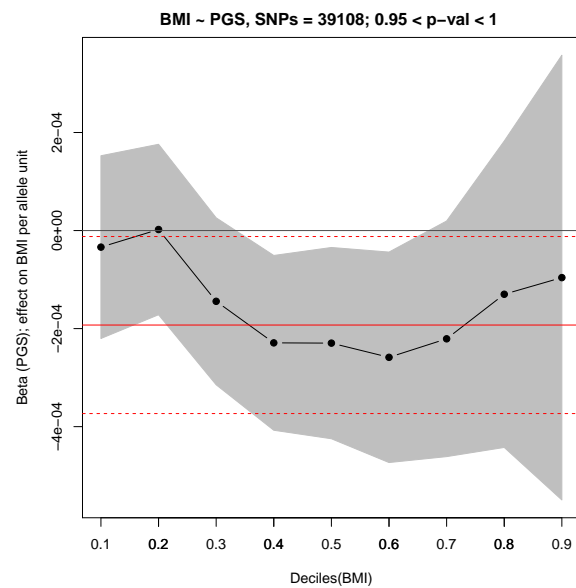

Supplement: Supplementary file 1 [file genes-09-00411-s001.zip › SupplementaryFigure2.pdf]

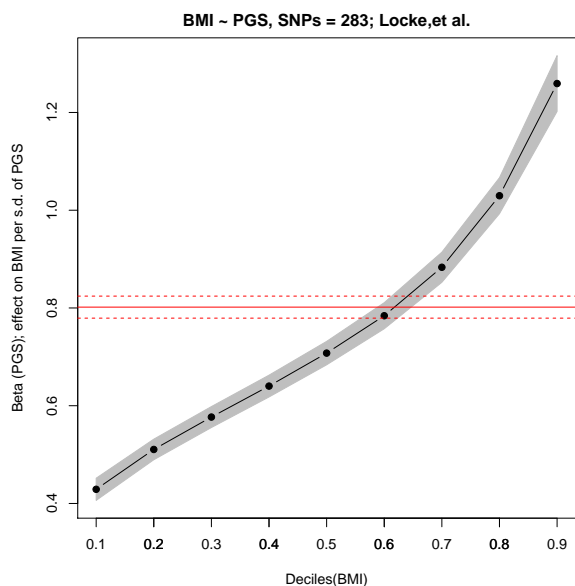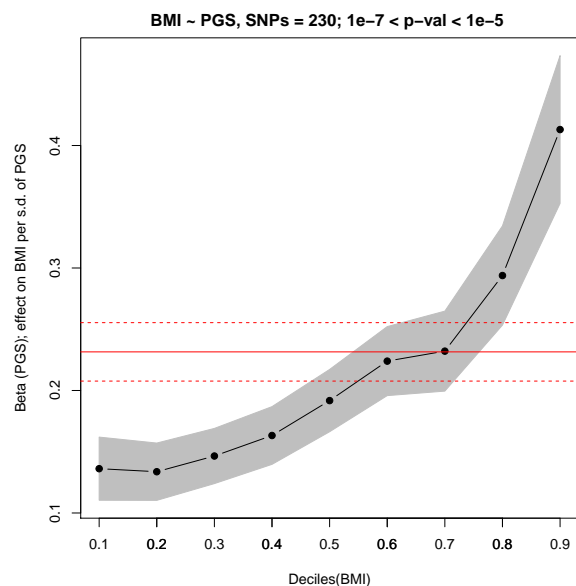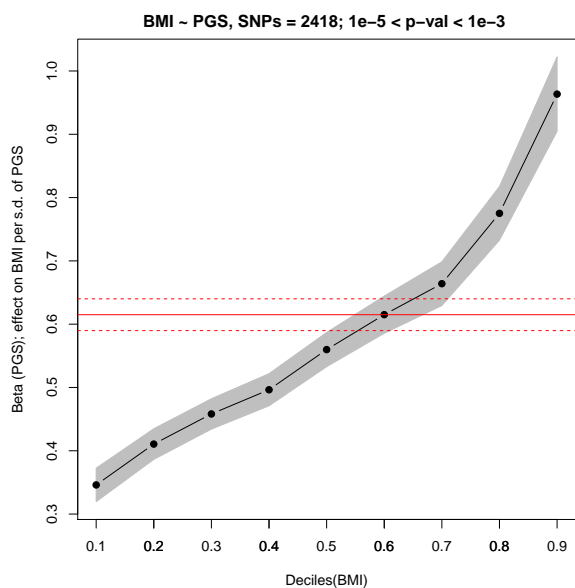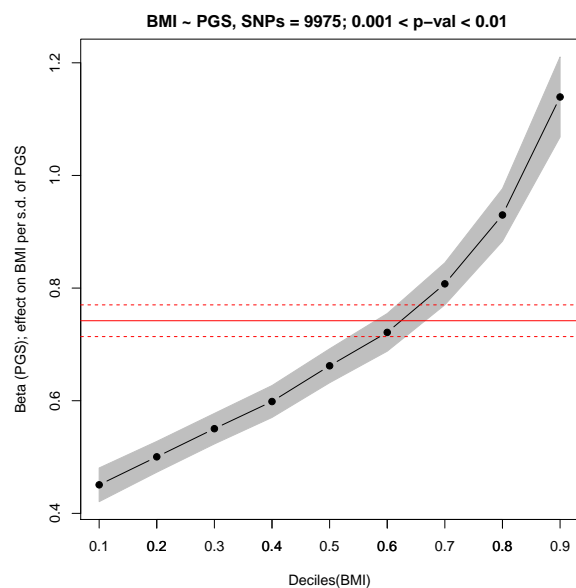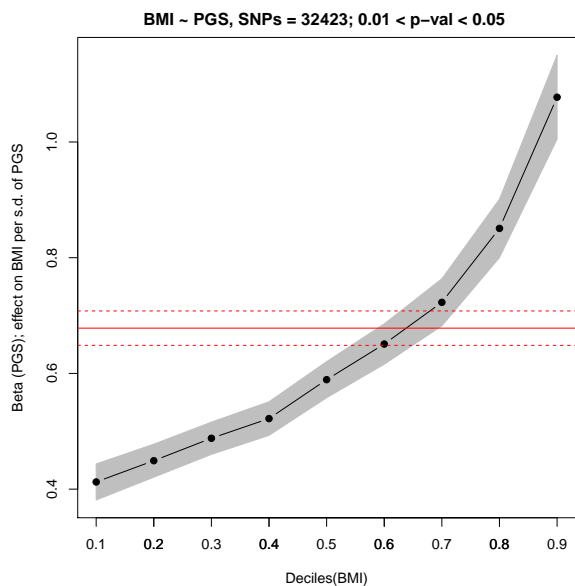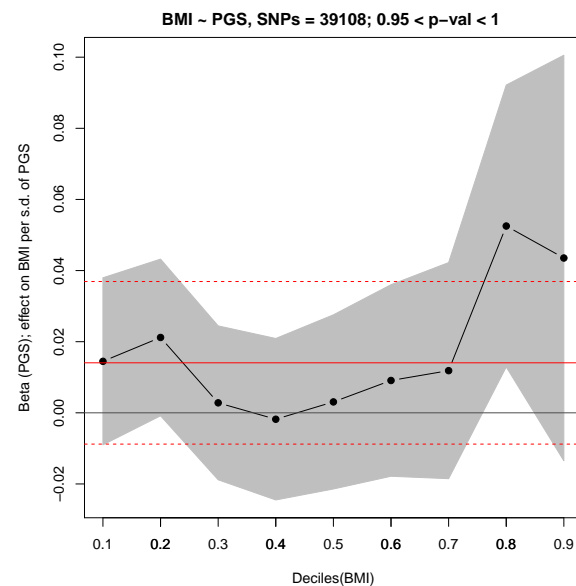

Supplement: Supplementary file 1 [file genes-09-00411-s001.zip › SupplementaryFigure3.pdf]

A

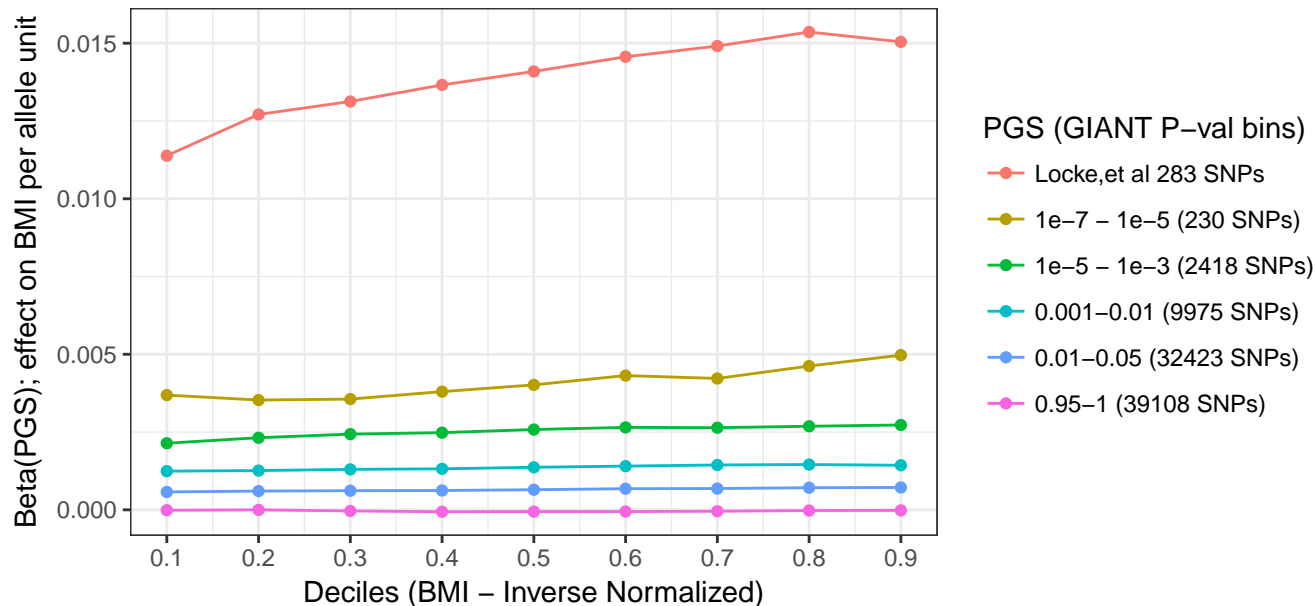

B

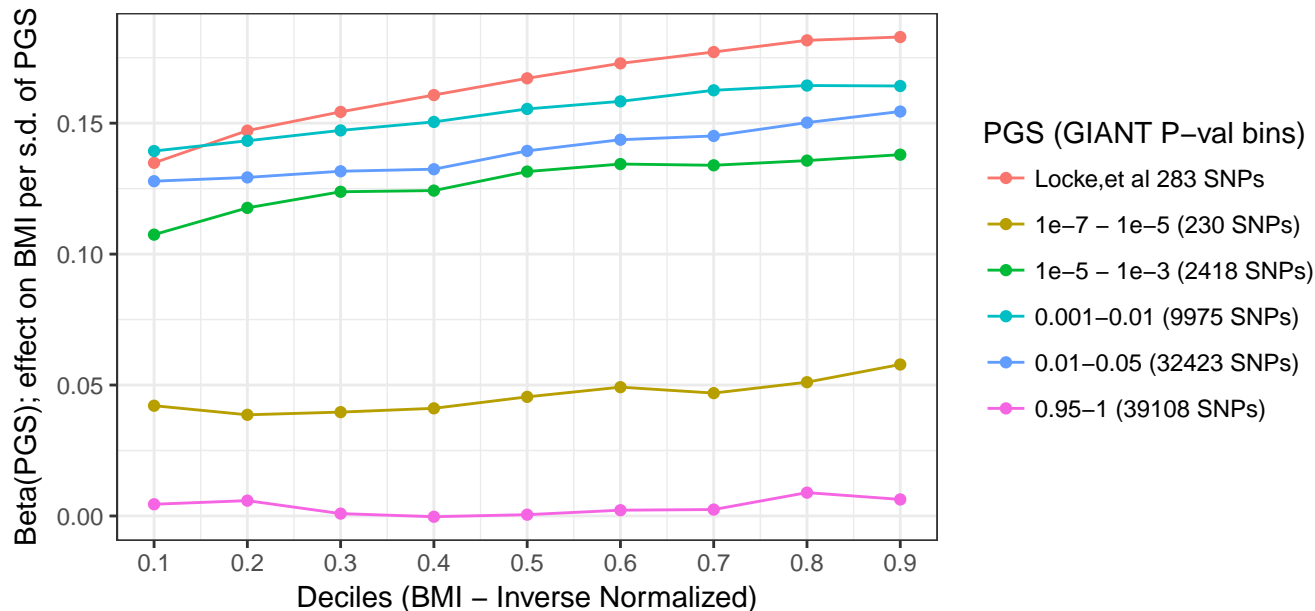

Supplement: Supplementary file 1 [file genes-09-00411-s001.zip › SupplementaryFigure4.pdf]

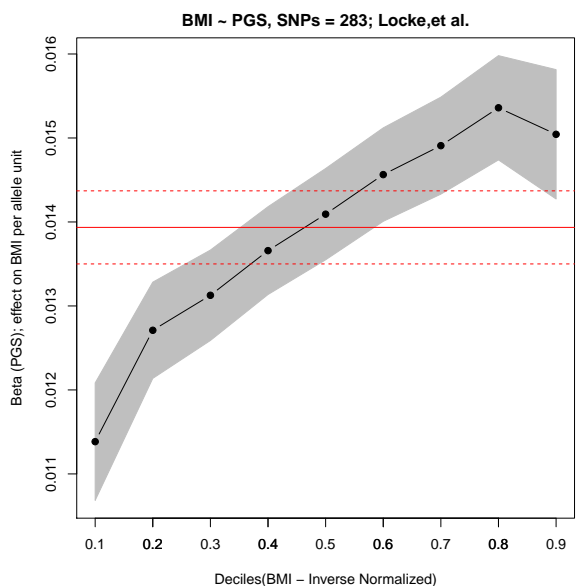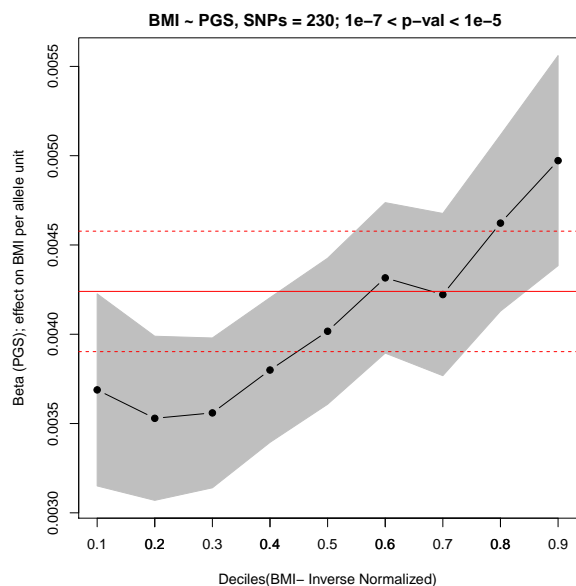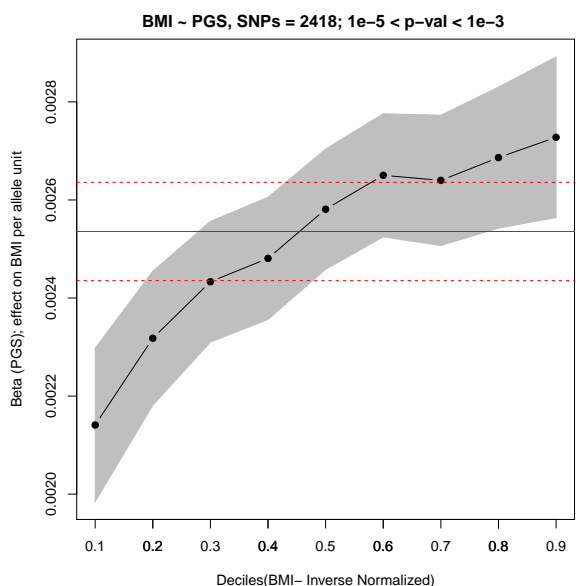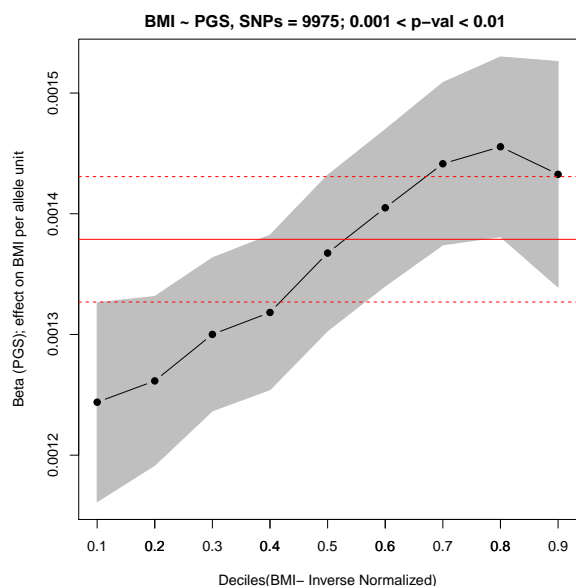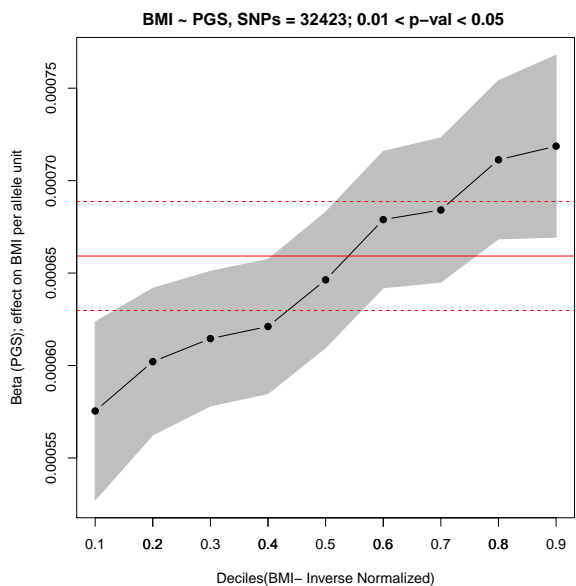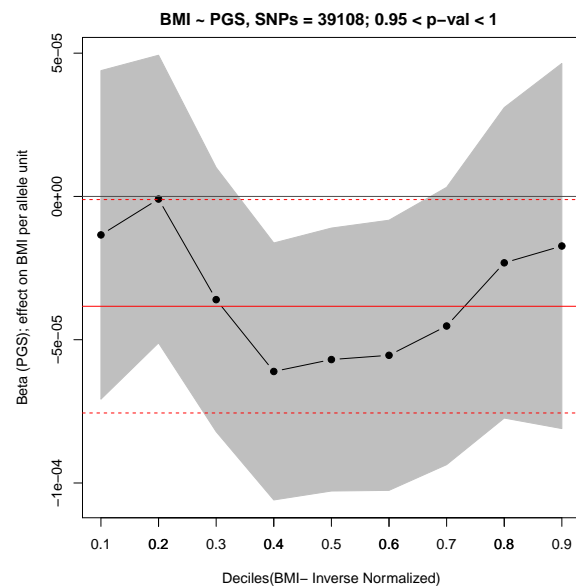

Supplement: Supplementary file 1 [file genes-09-00411-s001.zip › SupplementaryFigure5.pdf]

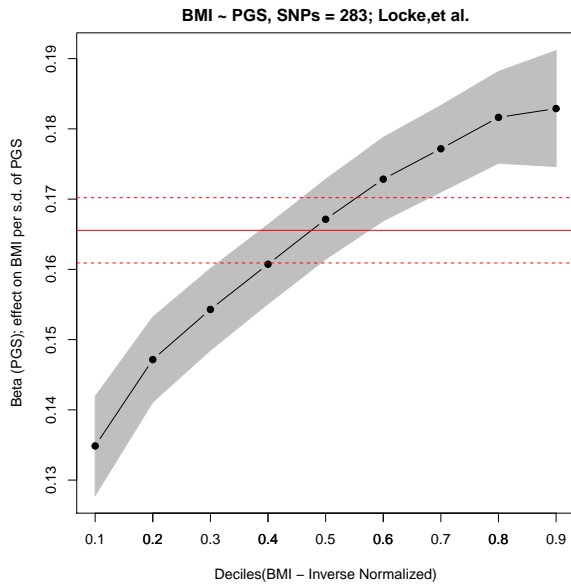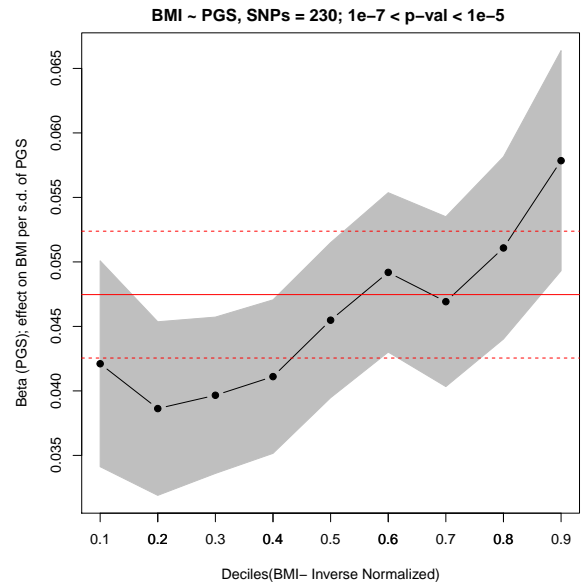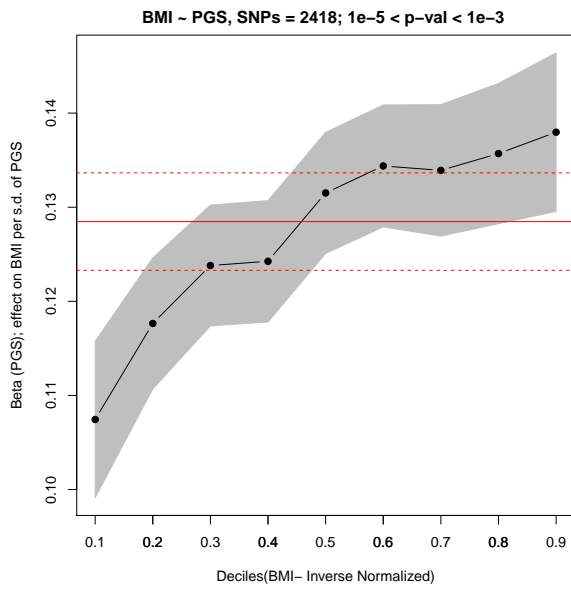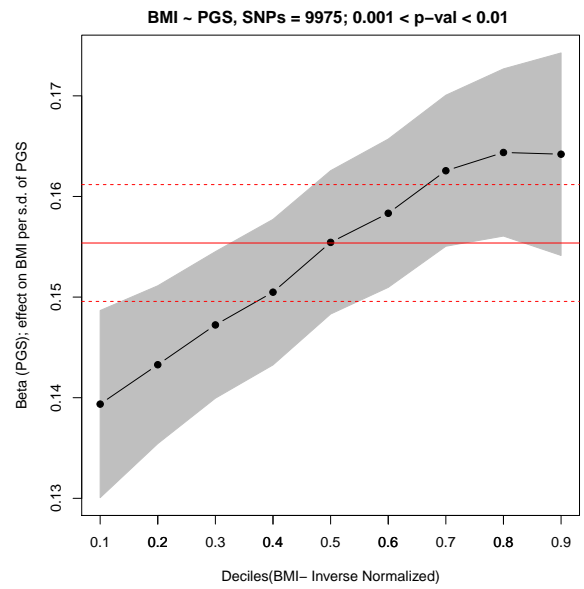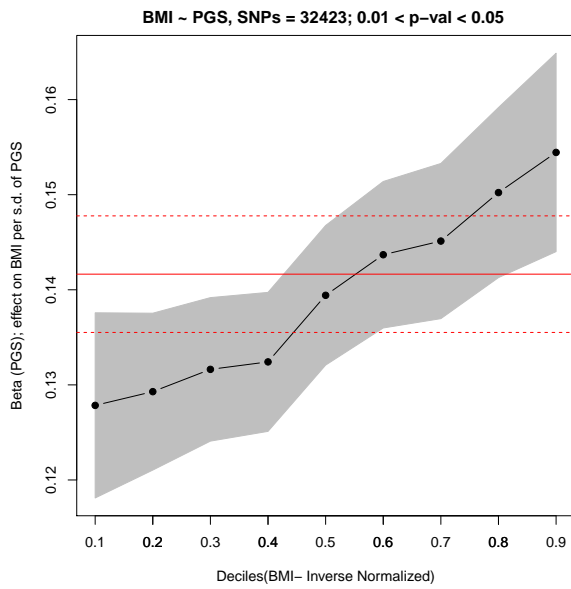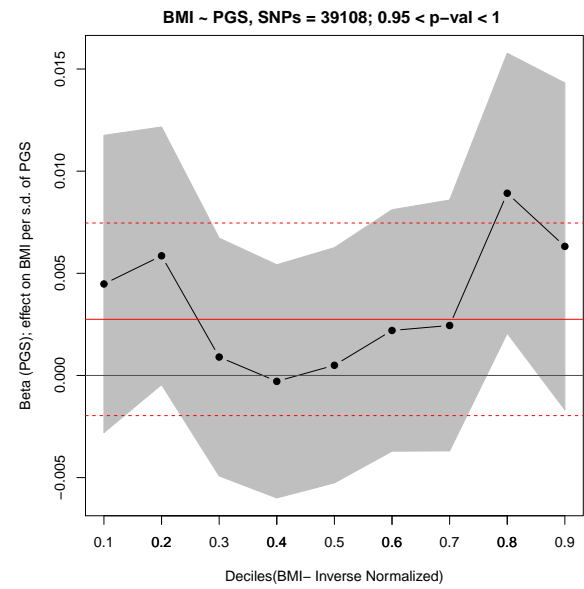

Supplement: Supplementary file 1 [file genes-09-00411-s001.zip › SupplementaryFigure6.pdf]
